# Uncoupling Lipid Synthesis from Adipocyte Development

**DOI:** 10.3390/biomedicines11041132

**Published:** 2023-04-09

**Authors:** Qianfen Wan, Carmen Calhoun, Tarik Zahr, Li Qiang

**Affiliations:** 1 Naomi Berrie Diabetes Center, Columbia University, New York, NY 10032, USA; 2 Department of Pathology and Cell Biology, Columbia University, New York, NY 10032, USA; 3 Molecular Pharmacology and Therapeutics, Columbia University, New York, NY 10032, USA

**Keywords:** obesity, adipocyte, adipogenesis, lipogenesis, fatty acid, ACC, FASN, DGAT

## Abstract

Obesity results from the expansion of adipose tissue, a versatile tissue regulating energy homeostasis, adipokine secretion, thermogenesis, and inflammation. The primary function of adipocytes is thought to be lipid storage through lipid synthesis, which is presumably intertwined with adipogenesis. However, during prolonged fasting, adipocytes are depleted of lipid droplets yet retain endocrine function and an instant response to nutrients. This observation led us to question whether lipid synthesis and storage can be uncoupled from adipogenesis and adipocyte function. By inhibiting key enzymes in the lipid synthesis pathway during adipocyte development, we demonstrated that a basal level of lipid synthesis is essential for adipogenesis initiation but not for maturation and maintenance of adipocyte identity. Furthermore, inducing dedifferentiation of mature adipocytes abrogated adipocyte identity but not lipid storage. These findings suggest that lipid synthesis and storage are not the defining features of adipocytes and raise the possibility of uncoupling lipid synthesis from adipocyte development to achieve smaller and healthier adipocytes for the treatment of obesity and related disorders.

## 1. Introduction

The remarkable capacity of adipose tissue (AT) to store excess energy in the form of triglycerides (TG) via lipid synthesis has evolved as a lifesaving adaptation. Now, a high-calorie, sedentary environment has led to an epidemic of overweight and obesity, which have become leading health-related risk factors [1]. Historically, AT was a relatively neglected field of interest due to its supposed functional simplicity and inertness in metabolic regulation [2]. However, advancements in the past three decades have revealed AT to be the largest human endocrine organ and to be involved in immune activity. Dysregulation of these functions can lead to systemic alterations in insulin sensitivity, inflammation, and atherosclerosis, which are associated with major health conditions, such as type 2 diabetes (T2D) and cardiovascular disease (CVD) [3,4,5,6,7,8,9].

Adipocytes exert their endocrine effect upon distant tissues and neighboring cells via the release of signaling proteins known as adipokines. For example, leptin is known to affect satiety and feeding behavior controlled by the brain [10,11,12,13]. Adipsin (also known as Complement Factor D (Cfd)) can alter β cell functioning in the pancreas [14] and regulate bone remodeling [15]. Direct interactions between adipocytes and immune cells reciprocally affect adipocyte functioning, AT development, metabolic sensing, and inflammation [16,17,18,19]. The bidirectional feedback of AT with endocrine and immune functions reveals many intricate channels through which AT communicates systemic homeostatic conditions.

Of the myriad roles of AT, the primary functions of white adipocytes are commonly perceived to be lipid synthesis, including de novo lipogenesis of fatty acid (FA) and subsequent TG synthesis and lipid storage in lipid droplets (LDs). Under calorie excess, energy storage is achieved through two mechanisms of AT expansion, hyperplasia, and hypertrophy, which both result in weight gain but have different effects on systemic functioning. In hyperplasia, expansion is achieved by increasing the number of adipocytes through adipogenesis, the differentiation of adipocyte precursors into mature adipocytes. During hypertrophy, lipid droplets and adipocytes expand via lipid synthesis. Excessive hypertrophic AT expansion is a driving force for macrophage infiltration, chronic inflammation, and insulin resistance, which are accompanied and exacerbated by decreased adipogenesis, resulting in lipid deposition in more metabolically harmful places, such as visceral adipose depots and ectopic tissues (e.g., liver, heart, and skeletal muscle) [3,20,21]. Multiple studies show that inhibition of lipid synthesis, including targeting on ACCs [22,23,24,25,26], FASN [27,28,29,30], or DGATs [31,32,33,34] – results in beneficial effects on body weight loss, insulin sensitization, and prevention of nonalcoholic steatohepatitis. Therefore, controlling hypertrophic AT expansion via lipid synthesis inhibition is a viable therapeutic approach to alleviate the comorbidities of obesity. Reducing hypertrophy would entail reducing lipid synthesis and LD enlargement while maintaining or promoting adipogenesis. In order to achieve this goal, adipocyte development and function must be more clearly elucidated. Previous studies suggest adipokine secretions in white adipose tissue and cold-induced thermogenesis in brown adipose tissue could be uncoupled from TG synthesis [31,35]. This study investigates whether lipid synthesis and/or storage are necessary for adipocyte formation or maintenance of adipocyte identity. 

Adipogenesis can be divided into three phases: fate determination, differentiation, and hypertrophic growth [36,37,38]. During the first phase of fate determination, a pluripotent mesenchymal stem cell (MSC) commits to an adipocyte lineage, becoming a pre-adipocyte. Although no morphological changes occur, the pre-adipocyte has lost the ability to become other cells, such as myocytes, osteocytes, or chondrocytes. During the second phase of differentiation, the pre-adipocyte differentiates into an immature adipocyte. This process features morphological changes such as small LDs and also the development of functional features such as insulin sensitivity, adipokine secretion, and lipid transport and synthesis [39]. These changes are initiated and sustained by a central adipogenic cascade, in which C/EBPβ (CCAAT/enhancer binding protein β) activates PPARγ (peroxisome proliferator-activated receptor γ), which then activates C/EBPα (CCAAT/enhancer-binding protein α) [37,39]. The small adipocyte further accumulates TGs and eventually develops into a typical adipocyte with large LDs at the hypertrophic growth stage. Thus, adipogenesis is presumed to be essentially intertwined with lipid synthesis. Lipid synthesis is initiated in early adipogenesis and highly activated during hypertrophic growth. When considering harnessing AT expansion for obesity treatment, the uncoupling of lipid synthesis from adipogenesis appears to be a plausible strategy, especially in the later stage of hypertrophic growth. 

In the present study, we found that AT from mice under prolonged fasting loses lipid-containing morphology but still maintains adipocyte function, including adipokine production and rapid nutrient responses. This observation prompted us to consider the potential for adipocyte differentiation, identity, and functioning to be uncoupled from lipid synthesis and storage. To test this notion, we inhibited several crucial enzymes in the lipid synthesis pathway at different stages of adipogenesis and also induced adipocyte dedifferentiation. Our findings demonstrate that a basal level of lipogenesis is essential for supporting adipocyte differentiation but not for maintaining adipocyte identity or function after maturation. Therefore, lipid synthesis is not inextricably intertwined with adipogenesis. The uncoupling of lipid synthesis from adipogenesis not only deepens our understanding of adipocyte biology but also highlights a promising direction for obesity management.

## 2. Materials and Methods

### 2.1. Animal Studies

The 10-wk-old C57BL/6J male mice were housed at 23 ± 1 °C on a 12-h light/12-h dark cycle with free access to a normal chow diet and water. Mice were separated into three groups, including an *ad libitum* feeding group (constant access to chow diet), 72-h fasting group, and 72-h fasting followed by 3 h chow diet refeeding group. The animal study was approved by Columbia University Animal Care and Utilization Committee.

### 2.2. Cell Culture and Adipocyte Differentiation

3T3-L1 and C3H10T1/2 mouse adipocyte cell lines were purchased from American Type Culture Collection (ATCC) and cultivated in DMEM high glucose medium supplemented with 10% FCS (for 3T3-L1) or 10% FBS (for C3H10T1/2) and 1% Penicillin-Streptomycin. After two days post-confluence, differentiation was induced in 3T3-L1 cells by adding a differentiation cocktail containing 0.5 mM 3-Isobutyl-1-methylxanthine (IBMX), 1 µM Dexamethasone, and 5 µg/mL insulin. Cell medium was removed and switched to differentiation maintenance medium containing 5 µg/mL insulin after three days post-differentiation. For C3H10T1/2 adipocyte differentiation, a similar method was used, except with additional Rosiglitazone (Rosi) (5 µM) in the differentiation-inducing cocktail from differentiation Day 0 to Day 3.

The PPARγ2 stably-expressed mouse embryonic fibroblast (MEF) cell line (PPARγ OE MEF) was generated and validated previously [40,41]. The differentiation protocol is the same as in previous studies [40,41,42]. In detail, two days post-confluence, cells were incubated with the same differentiation cocktail as used in 3T3-L1 cells differentiation with additional Rosi (5 µM) and doxycycline (3.3 µg/mL). After three days of differentiation, the cell medium was replaced with a differentiation maintenance medium containing Rosi (5 µM), 5 µg/mL insulin, and doxycycline (3.3 µg/mL).

### 2.3. Adipocyte Treatments

Avidin treatment: 3T3-L1 and C3H10T1/2 cells were treated with Avidin (10^−7^ M) at the indicated time during differentiation. Cells were harvested for gene expression analysis and Oil Red O staining.

FASN inhibitor treatment: 3T3-L1, C3H10T1/2, and PPARγ2 OE MEF cells were treated with the FASN inhibitor TVB-3664 (200 nM) from differentiation Day 0 to Day 6 or Day 3 to Day 6. In addition, 3T3-L1 and C3H10T1/2 mature adipocytes were treated with TVB-3664 (200 nM) from differentiation Day 6 to Day 9. Cells were harvested for gene expression analysis and Oil Red O staining.

DGAT1/2 inhibitor treatment: 3T3-L1 or C3H10T1/2 cells were treated with the DGAT1/2 inhibitors PF-04620110 (3 µM) and PF-06427878 (3 µM) from differentiation Day 0 to Day 6. Cells were harvested for gene expression analysis and Oil Red O staining.

TGF-β treatment: Mature C3H10T1/2 adipocytes were treated with TGF-β (5 ng/mL) from differentiation Day 6 to Day 12. Cells were harvested for gene expression analysis and Oil Red O staining.

### 2.4. Oil Red O Staining

After the removal of the cell medium, cells were fixed with 10% formalin for at least 30 min and then incubated with 60% isopropanol for 5 min. Finally, cells were stained with Oil Red O solution for 15 min, then rinsed with water until the water was clear. Stained cells were photographed under a microscope. Oil Red O staining was further quantified by dissolving Oil Red O in an isopropanol solution for 10 min, and then absorbance was read at 492 nm.

### 2.5. Gene Expression Analysis

After removing the culture medium, cells in a 6-well plate were gently rinsed with cold PBS and then performed RNA isolation using IBI Tri-isolate total RNA kits (IBI Scientific, Dubuque, IA, USA). Tissues were homogenized and then performed RNA purification using the same RNA isolation kit. High-capacity cDNA Reverse Transcription kit (Applied Biosystems, Foster City, CA, USA) was used to generate complementary DNA (cDNA) from a 1000 ng RNA template. Quantitative real-time PCR (Q-PCR) was performed on Bio-Rad CFX96 Real-Time PCR system by using AzuraView™ Green Fast qPCR Blue Mix (Azura Genomics, Raynham, MA, USA). The PCR program was set to initial denaturation at 95 °C for 2 min, then denature at 95 °C for 5 s, anneal, and extension at 64 °C for 30 s for a total of 40 cycles. The relative gene expression fold change was evaluated using the ∆∆Ct method. *Rpl23* or *Cpa* was used as the reference genes. The primer sequences are listed in Supplementary Table S1.

### 2.6. Protein Analysis

Plasma samples were diluted in PBS (phosphate-buffered saline) 100 times. The same amount of diluted plasma sample was loaded for western blotting analysis. Antibodies of Adipsin (R&D Systems, Minneapolis, MN, USA; catalogue AF5430) and Adiponectin (Thermo Fisher Scientific, Waltham, MA, USA, catalogue PA1-054) were used in this study. The band intensity was quantified using ImageJ (v. 2.1.0; National Institutes of Health). 

### 2.7. Histological Assessments and Immunohistochemistry

The tissues were removed and fixed in 10% formalin, embedded in paraffin, and stained with Hematoxylin and Eosin (H&E). The procedure of immunohistochemical staining was described in a previous study [43]. Adipose tissue sections were incubated with the antibody of Perilipin 1 (PLIN1, CST #9349) and Adipsin (CFD, R&D Systems, # AF5430) at a 1:200 dilution in PBST (Phosphate Buffered Saline with Tween 20) overnight, respectively.

### 2.8. Statistical Analysis

Unpaired 2-tailed Student’s *t*-tests were used to evaluate the significance between groups. The standard for a significant difference between groups was *p* < 0.05. All values were represented as mean ± standard error of the mean (SEM). Sample sizes are included in the figure legends.

## 3. Results

### 3.1. Prolonged Fasting Leads to “Adipocyte Dormancy”

**Figure 1:**
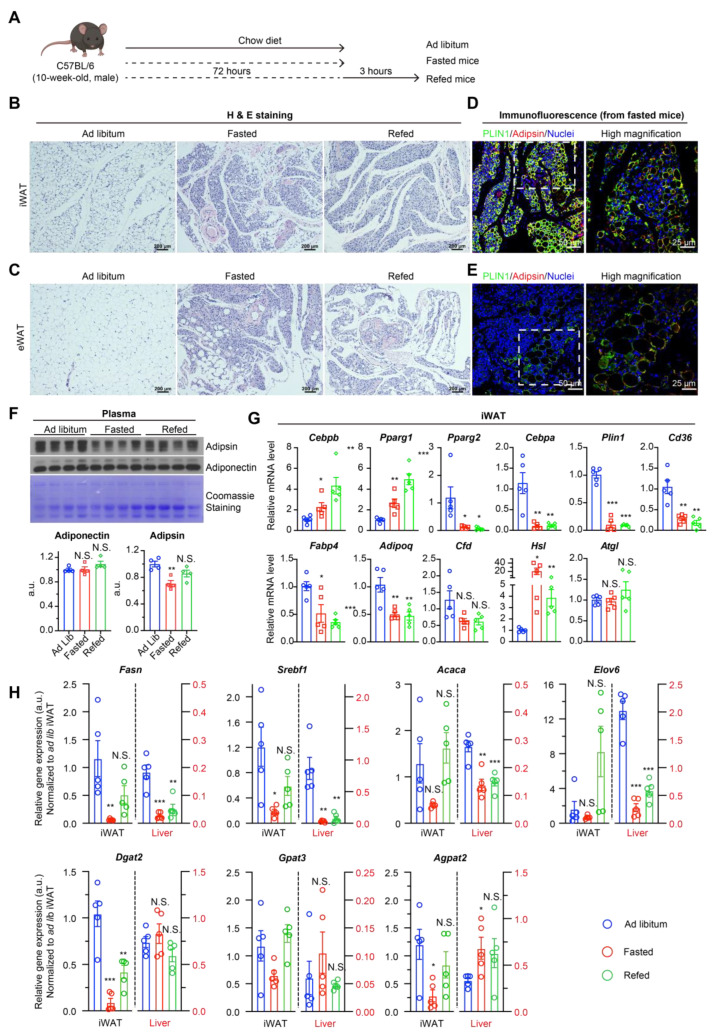
Prolonged fasting deprives lipid storage but adipocyte identity in mice. (**A**): Experimental design. Chow diet-fed C57BL/6 mice were subjected to 72-h fasting with or without 3 h refeeding. (**B**,**C**,**D**,**E**) staining of iWAT (**B**) and eWAT (**C**) from *ad libitum* fed, fasted mice or fasted-refed mice. (**D**,**E**): Confocal immunofluorescence of iWAT (**D**) and eWAT (**E**) from fasted mice for indicated proteins. (**F**): Western blotting analysis of plasma adipokines. (**G**,**H**): qPCR analysis of gene expression of adipogenic and lipolytic markers (**G**) and synthetic lipid genes (**H**) in iWAT from *ad libitum* fed, fasted mice, or fasted-refed mice. The fold changes for gene expression in iWAT (Y-axis on the left side of the graph) and liver (Y-axis on the right side of the graph) were normalized to its expression in iWAT from mice on *ad libitum* condition. n = 5/group. Data were represented as mean ± SEM. Statistical significance was calculated via two-tailed Student’s *t*-tests (Fasted or refed group versus *ad libitum* group). N.S.: not significant, * *p* < 0.05, ** *p* < 0.01, *** *p* < 0.001.

Prolonged fasting of mice was used as a naturally occurring method to halt lipid synthesis and indirectly induced LD-deprived adipocytes (Figure 1A). After 72 h of prolonged fasting, fat mass was drastically reduced to meet energy demands. The H&E-stained sections of inguinal white adipose tissue (iWAT) and epididymal white adipose tissue (eWAT) showed substantial loss of adipocyte morphology with few dispersed LD-containing adipocytes remaining (Figure 1B,C). In contrast, the morphological changes in liver and brown adipose tissue (BAT) were much milder, though BAT showed less lipid accumulation in the fasting state accompanied by modest changes in thermogenic gene expression (Supplementary Figure S1A,B). As an LD-coating protein, Perilipin 1 (PLIN1) is restricted to adipocytes. While a strong Perilipin 1 signal was detected in preserved adipocytes containing large LDs, a weaker fluorescent signal was observed in cells with non-visualizable LD after prolonged fasting in both depots (Figure 1D,E). Interestingly, the fluorescent signal of adipsin, a white adipocyte-specific adipokine, was consistent across cells with and without LDs (Figure 1D,E). Moreover, the levels of Adiponectin and Adipsin, two representative adipokines, had minor changes in the circulation (Figure 1F), reinforcing adipocyte endocrine function as operating independently of TG storage. Underpinning fat mass loss is the prevalent repression of mature adipocyte markers (e.g., *Cebpa*, *Pparg2*, *Plin1*, and *Cd36*) and key lipogenic genes (e.g., *Fasn*, *Srebf1*, *Acaca*, *Elov6*, *Gpat3*, *Agpat2*, and *Dgat2*), as expected during a catabolic, nutrient-deprived state (Figure 1G,H). In contrast to the dramatic repression of these genes, the expression of adipokine genes (e.g., *Fabp4*, *Adipoq*, and *Cfd*) was only moderately (~50%) decreased, whereas the early adipogenic transcription factors *Cebpb* and *Pparg1* were up-regulated after fasting. Lipolytic gene *Hsl* was markedly induced by fasting while *Atgl* remained constant (Figure 1G). Together, the morphological and gene expression data imply that adipocytes can enter a dormant state deprived of lipid storage while preserving endocrine function. 

Response to refeeding revealed the metabolic plasticity of adipocytes that maintains their functional identity after a period of food scarcity. After 3-h refeeding, the synthetic lipid genes were mostly restored (Figure 1H), although visible LDs did not form because of the limited refeeding period (Figure 1B,C). We included liver tissue as a comparison for lipid synthesis activity and nutrient response, given that the liver is another major site of lipid synthesis. Both AT and liver revealed similar responses to prolonged fasting regarding lipogenic genes (*Fasn*, *Srebf1*, *Acaca*, and *Elovl6*) but not TG synthesis genes (*Gpat3*, *Agpat2*, and *Dgat2*) (Figure 1H). However, under the *ad libitum* condition, AT expression levels of nearly all lipid synthetic genes were higher—often by several folds—as compared to respective liver equivalents. Furthermore, the restoration of lipogenic gene expression by refeeding in AT was not seen in the liver. Both the higher basal levels of lipid synthetic genes and higher responsiveness to refeeding indicate that AT is the primary lipid synthesis and lipid storage organ with the capacity to rapidly uptake nutrients. In contrast, all adipogenic genes except the early adipogenic factors *Cebpb* and *Pparg1* remained low under the 3-h refeeding condition (Figure 1G), suggesting that adipogenic gene expression is not always associated with adipocyte identity and/or that adipocytes enter a period of “dormancy” during nutrient deprivation. The maintenance of adipocyte identity may allow for rapid response to nutrients. As such, the retention of adipocyte identity and functioning despite suppressed lipid synthesis during prolonged fasting, in addition to the rapid response in lipid synthetic genes during refeeding, demonstrates the potential to uncouple lipid synthesis from adipocyte identity. 

### 3.2. Biotin Deprivation—First Glimpse of Uncoupling

**Figure 2:**
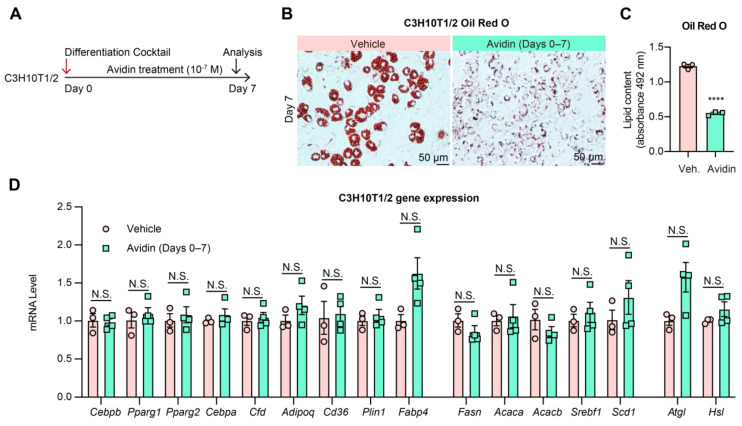
Biotin deprivation causes adipogenesis without TG accumulation. (**A**): Experimental design. C3H10T1/2 pre-adipocytes were differentiated in the presence of 10^−7^ M Avidin from Day 0 to Day 7 of differentiation. (**B**): Oil Red O staining of lipid droplets in C3H10T1/2 cells on Day 7 of differentiation. (**C**): Quantification of Oil Red O staining (n = 3, 3). (**D**): qPCR analysis of gene expression of adipogenic markers and lipogenic genes on Day 7 of differentiation with or without Avidin treatment. (n = 3, 4). Data were represented as mean ± SEM. Statistical significance was calculated via two-tailed Student’s *t*-tests. N.S.: not significant, **** *p* < 0.0001.

Given the separate regulation of lipid synthesis and adipocyte functioning under natural conditions, several enzymes along the lipid synthesis pathway were inhibited in vitro to determine the potential to uncouple lipid synthesis from adipocytes. First, we targeted the initial enzyme of the de novo lipogenesis pathway: acetyl CoA carboxylase (ACC1 and ACC2), a biotin-dependent carboxylase. An early study found that 3T3-L1 pre-adipocytes maintained the ability to differentiate into adipocytes when using biotin-deficient media, but these newly formed adipocytes did not accumulate LDs [44]. In the present study, Avidin, a strong biotin-binding protein, was used to block biotin functioning during the differentiation of C3H10T1/2 cells and 3T3-L1 cells and examine the potential uncoupling of lipogenesis, adipogenesis, and adipocyte identity (Figure 2A, Supplementary Figure S2A). These two classic adipogenesis models showed a consistent reduction in LD formation under Avidin treatment, although adipocyte differentiation efficacy and the change of morphology were maintained (Figure 2B,C, Supplementary Figure S2B,C). No changes in adipogenic, lipid synthetic, or lipolytic gene expression were observed in either cell line (Figure 2D, Supplementary Figure S2D). Moreover, the canonical adipokine genes *Adipoq* and *Cfd* were expressed at normal levels, indicating intact adipocyte endocrine function during lipid storage deficiency. Interestingly, direct inhibition of ACC1/2 by soraphen A inhibits both early adipogenesis and lipid accumulation in 3T3-L1 differentiation [45]. This difference in uncoupling from biotin deprivation is likely due to the complete inhibition of ACC1/2 activity by soraphen A compared to a possibly incomplete biotin deprivation by avidin which likely allowed for a residual functioning of ACC1/2 to supply a basal lipogenesis for subsequent differentiation. Therefore, a basal flux of the lipogenesis pathway likely serves as a checkpoint for pre-adipocytes to enter differentiation. Given this, lipogenesis and lipid storage can be significantly decreased and uncoupled from adipocyte development and endocrine function. 

### 3.3. Fatty Acid Synthase Inhibition—Basal Fatty Acid Synthesis Is Coupled to Early Adipogenesis

**Figure 3:**
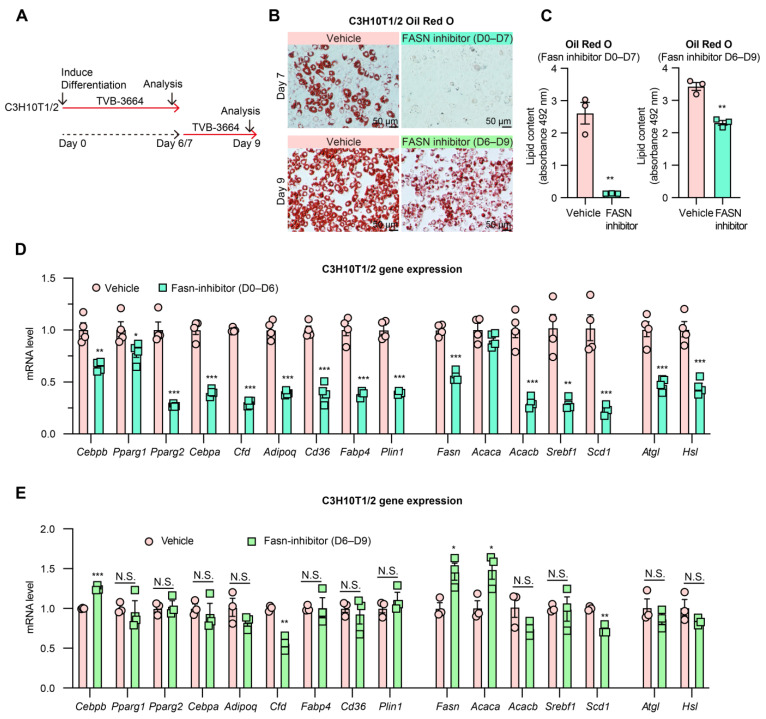
Inhibition of FASN shows a stage-dependent effect on adipogenesis. (**A**): Experimental design. C3H10T1/2 pre-adipocytes were treated with FASN inhibitor TVB-3664 (200 nM) from the beginning of differentiation Day 0 to Day 6/7 or from Day 6 to Day 9. (**B**): Oil Red O staining of lipid droplets in C3H10T1/2 cells (fixed on Day 7 or Day 9 with or without TVB-3664 treatment). (**C**): Quantification of Oil Red O staining. (**D**): Gene expression of adipogenic markers and lipogenic genes in C3H10T1/2 cells were treated with TVB-3664 from Day 0 to Day 6 during differentiation (n = 4, 4). (**E**): Gene expression of C3H10T1/2 cells after TVB-3664 treatment from Day 6 to Day 9 (n = 3, 3). N.S.: not significant, * *p* < 0.05, ** *p* < 0.01, *** *p* < 0.001 for control vs. TVB-3664 treatment group by 2-tailed Student’s *t*-test. Data were represented as mean ± SEM.

Next, we inhibited the rate-limiting enzyme fatty acid synthase (FASN) in the FA synthesis pathway to investigate whether the inhibition of lipogenesis through targeting different enzymes could have a similar uncoupling effect on adipogenesis. C3H10T1/2 and 3T3-L1 cells were treated with a potent FASN inhibitor TVB-3664 during differentiation (Days 0–6 or 7) and after differentiation (Days 6–9) (Figure 3A, Supplementary Figure S3A). Treatment throughout differentiation (Days 0–6 or 7) resulted in a complete block of lipid accumulation accompanied by a dramatic decrease in markers of both adipogenesis, lipogenesis, and lipolysis (Figure 3B–D, Supplementary Figure S3B–D), in agreement with a previous study using different inhibitors to block FASN activity [46]. However, treatment of mature adipocytes (Days 6–9) resulted in only minor changes in adipogenic gene expression (Figure 3E, Supplementary Figure S3E–G). LD formation was also reduced (Figure 3B,C), although to a lesser extent, while changes in lipogenic genes reflected feedback inhibition or stimulation of the pathway according to where intermediates would respectively decrease or accumulate due to FASN inhibition. The difference in adipogenic gene expression between treatments of the different stages suggests that lipogenesis is most essential to early adipocyte differentiation and much less necessary for maintaining adipocyte identity during the hypertrophic growth stage.

### 3.4. PPARγ Activation Fails to Rescue the Impaired Adipogenesis by FASN Inhibition

**Figure 4:**
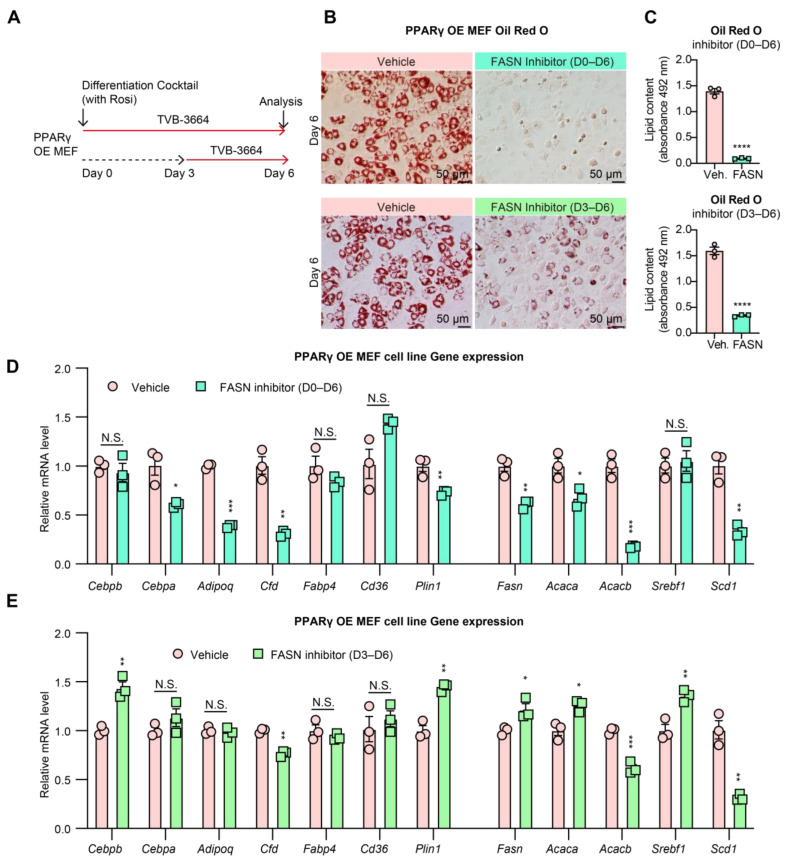
PPARγ2 cannot rescue adipogenesis from FASN inhibition. (**A**): Experimental design. PPARγ2 OE MEF cells were treated with the FASN inhibitor TVB-3664 (200 nM) from differentiation Day 0 to Day 6 or Day 3 to Day 6. (**B**): Oil Red O staining of lipid droplets of differentiated PPARγ2 OE MEF cells (fixed on Day 6 with or without treatment). (**C**): Quantification of Oil Red O staining (n = 3, 3). (**D**,**E**): Gene expression of PPARγ2 OE MEF cells treated with TVB-3664 at Day 0 to Day 6 (**D**) or Day 3 to Day 6 (**E**) during differentiation (n = 3, 3). N.S.: not significant, * *p* < 0.05, ** *p* < 0.01, *** *p* < 0.001, **** *p* < 0.0001 for control vs. TVB-3664 treatment group by 2-tailed Student’s *t*-test. Data were represented as mean ± SEM.

As a master regulator of adipocyte development, PPARγ is essential for adipocyte differentiation and lipid accumulation. FASN inhibition during adipocyte differentiation dramatically suppressed PPARγ expression, particularly the dominant isoform *Pparg2* (Figure 3D). Furthermore, FASN produces fatty acid substrates used in the synthesis of PPARγ ligand [47]. Therefore, we considered whether decreased PPARγ expression and activity contributed to the impaired differentiation during FASN inhibition. In other words, we suspect that decreasing lipogenesis while increasing adipogenesis could be achieved by combining FASN inhibition with increased PPARγ levels and activity. To investigate this possibility, we stably overexpressed PPARγ2 in the *Pparg^−/−^* mouse embryonic fibroblasts (MEFs) [40] and stimulated full activation with the thiazolidinedione (TZD) agonist rosiglitazone (Rosi) while inhibiting FASN (Figure 4A). Treatment with FASN inhibitor TVB-3664 throughout differentiation (Days 0–6) still resulted in blocked lipid accumulation and impaired expression of mature adipocyte genes (*Cepba*, *Adipoq*, *Cfd*, and *Plin1*) and lipogenic genes, but not the canonical downstream genes targeted by PPARγ (*Fabp4* and *Cd36*) (Figure 4B–D). In contrast, when treatment was restricted to the later stage of differentiation (Days 3–6), adipogenic and adipokine genes demonstrated an almost complete recovery in expression (Figure 4E). However, LDs were greatly reduced, though still visible (Figure 4B). These findings further support lipogenesis as a requirement for early differentiation. FASN and de novo FA synthesis serve an essential role in differentiation outside of PPARγ expression and activation. These findings deviate from the dogma that PPARγ activation is necessary and sufficient to drive adipocyte differentiation.

### 3.5. DGAT1/2 Inhibition Leads to “Pseudo Uncoupling” of Lipid Synthesis and Adipogenesis

**Figure 5:**
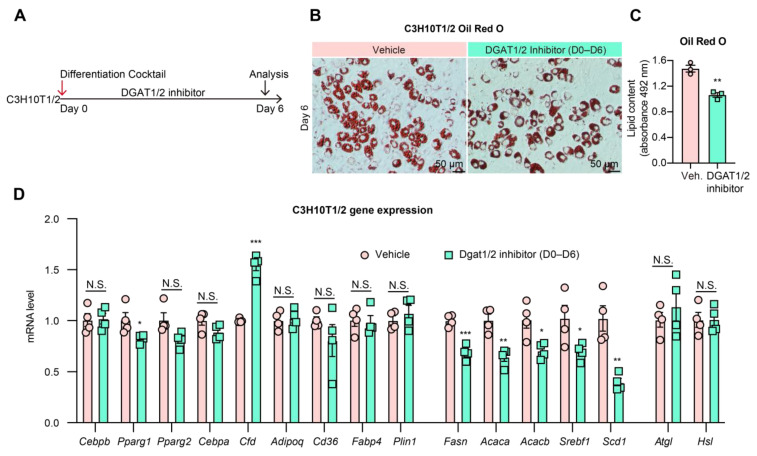
DGAT1/2 inhibition shows normal adipogenesis. (**A**): Experimental design. C3H10T1/2 pre-adipocytes were differentiated in the presence of DGAT1 inhibitor (PF-04620110, 3 µM) and DGAT2 inhibitor (PF-06427878, 3 µM) from Day 0 to Day 6. (**B**): Oil Red O staining of lipid droplets in C3H10T1/2 cells on Day 6 of differentiation. (**C**): Quantification of Oil Red O staining (n = 3, 3). (**D**): qPCR analysis of gene expression of adipogenic markers and lipogenic genes on Day 6 of differentiation (n = 4, 4). N.S.: not significant, * *p* < 0.05, ** *p* < 0.01, *** *p* < 0.001 for control vs. DGAT1/2 inhibitor treatment group by 2-tailed Student’s *t*-test. Data were represented as mean ± SEM.

As the final enzyme in TG synthesis, DGAT1/2 inhibition prevents only the synthesis of TGs and, thus, LD formation but does not prevent flux through the lipogenesis pathway itself [48]. As FA synthesis is essential for adipocyte differentiation, we reasoned that adipogenesis would not be halted by DGAT1/2 inhibition. To test this hypothesis, C3H10T1/2 and 3T3-L1 pre-adipocytes were treated with DGAT1/2 inhibitors from Days 0–7 of differentiation, and the effect on morphology and gene expression was measured (Figure 5A, Supplementary Figure S4A). Cell morphology revealed a decrease in LDs (Figure 5B,C, Supplementary Figure S3B,C), in agreement with the previous study [48]. Likewise, adipogenic and lipolytic genes’ expression remained constant in both cell lines and was even increased for 3T3-L1 cells (Figure 5D, Supplementary Figure S4D). Lipogenic gene expression was decreased in both cell lines, likely due to feedback inhibition from accumulated intermediates along the lipogenesis pathway. Together, these and previous findings support that neither TGs nor LDs are essential for adipocyte functioning or gene expression during differentiation. Instead, a basal level of de novo lipogenesis is of primary importance in initiating adipogenesis. However, DGAT1/2 inhibition results in normal adipogenesis but inhibits hypertrophic growth due to TG and LD absence, leading to a “pseudo-uncoupling” of adipogenesis and lipid synthesis since lipogenesis is still in flux. 

### 3.6. TGF-β Treated Mature Adipocytes Lose Adipocyte Identity but Maintain Lipid Droplets

**Figure 6:**
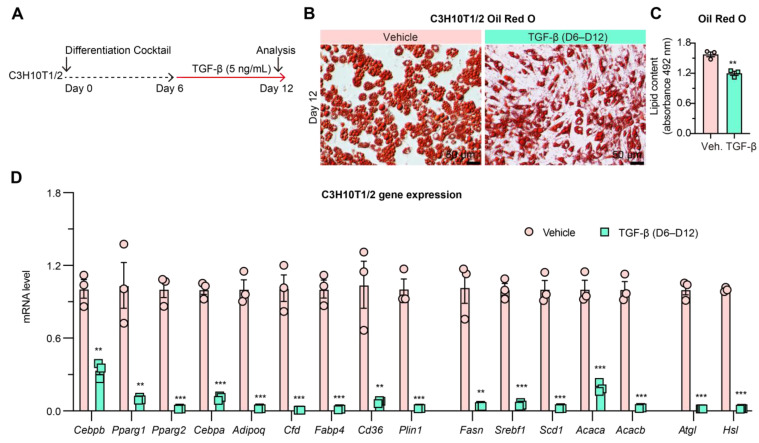
TGF-β induces adipocyte dedifferentiation. (**A**): Experimental design. Mature C3H10T1/2 cells were treated with TGF-β (5 ng/mL) from differentiation Day 6 to Day 12. (**B**): Oil Red O staining of lipid droplets in C3H10T1/2 cells on Day 12 of differentiation. (**C**): Quantification of Oil Red O staining (n = 3, 3). (**D**): qPCR analysis of gene expression in C3H10T1/2 cells on Day 12 of differentiation (n = 3, 3). ** *p* < 0.01, *** *p* < 0.001 for control vs. TGF-β group by 2-tailed Student’s *t*-test. Data were represented as mean ± SEM.

To further assess the uncoupling of lipid synthesis and adipogenesis, we implemented in vitro dedifferentiation of mature adipocytes by treating them with the pro-fibrotic cytokine transforming growth factor-β (TGF-β) (Figure 6A) [49,50]. The circular adipocyte morphology converted into a more linear, fibroblast-like cell structure, indicative of dedifferentiation (Figure 6B,C). Furthermore, TGF-β treatment dramatically repressed all adipogenic, lipogenic, and lipolytic genes in C3H10T1/2 adipocytes, suggesting loss of adipocyte identity (Figure 6D). Interestingly, LDs remained in cells despite the loss of adipocyte identity in a sort of “reverse uncoupling,” although this effect may have disappeared with longer treatment duration. The presence of LDs in dedifferentiated adipocytes further supports that adipocyte identity is not necessary for LD presence, as further supported in findings of ectopic fat infiltration into non-adipose tissues [51,52]. 

## 4. Discussion

**Figure 7:**
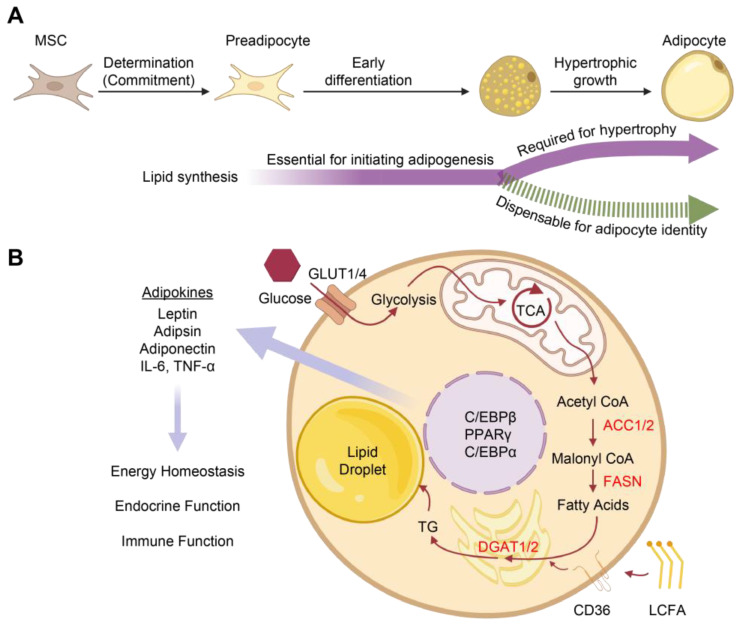
Lipid synthesis in adipocyte differentiation and functions. (**A**): The differentiation of mesenchymal stem cells (MSCs) into adipocytes begins with the first stage of fate determination, in which the cell commits to an adipocyte fate. Subsequently, the early stage of differentiation is accompanied by morphological and functional alterations and requires a basal level of lipogenesis. Lipid synthesis is required for the hypertrophic growth of adipocytes and adipocyte functions but is dispensable in the maintenance of adipocyte identity. (**B**): The lipid synthesis pathway and adipocyte functions. Abbreviations: ACC1/2, acetyl CoA carboxylase1/2; AT, Adipose tissue; C/EBPα, CCAAT/enhancer binding protein α; C/EBPβ, CCAAT/enhancer binding protein β; CD36, a cluster of differentiation 36; Adispin, complement factor D; DGAT1/2, diglyceride acyltransferase 1/2; LCFA, Long-chain fatty acid; FASN, fatty acid synthase; GLUT1/4, glucose transporter type 1/4; IL-6, interleukin 6; PPARγ, peroxisome proliferator-activated receptor γ; TG, triglyceride; TCA, tricarboxylic acid cycle; TNF-α, tumor necrosis factor-α.

Adipose tissue is not only the primary site of lipid storage but also an active endocrine organ integral to energy homeostasis regulation. We discovered that a basal level of lipogenesis is required for initiating adipocyte differentiation but not essential to maintaining adipocyte identity or function after maturation. Our findings indicate that lipid storage is not the defining feature of adipocytes, and that lipid synthesis can be conditionally uncoupled from adipogenesis, especially during the hypertrophic growth stage (Figure 7). Thus, our study deepens our understanding of adipocyte biology and offers the potential of a new approach to obesity intervention through uncoupling lipid synthesis from adipogenesis. 

The regulation and interaction of adipogenesis, lipogenesis, and adipocyte identity are closely entwined. In the conventional view, lipogenesis and adipogenesis are directly and inextricably coupled. However, they are distinct and show the potential to be uncoupled, especially when regulators more specific to each respective pathway are moderated. FA synthesis was seen to be of minor importance to the maintenance of adipocyte identity when the abundance and activity of PPARγ are compensated. However, FASN activity is necessary for early differentiation in functions outside of PPARγ activation as FAs not only function in cell membrane structure but are also involved in signaling transduction [53], protein lipidation [54], and more. The same is true for inhibiting ACC1/2. Biotin deprivation revealed that by targeting the cofactor of ACC1/2 in lipogenesis, adipocyte differentiation, and identity can be maintained while significantly reducing lipogenesis and LD formation. However, completely abolishing ACC1/2 activity with soraphen A blocks both differentiation and lipid storage [45]. The different outcomes may be due to the residual functioning of ACC1/2 in the incomplete biotin deprivation by avidin treatment, which allowed a basal level of FA synthesis to support early adipocyte differentiation. When inhibiting TG synthesis, our own and previous experiments of DGAT inhibition in vitro [48] and DGAT KO mice [31] demonstrate that the formation of TG and LDs is not necessary for normal adipocyte differentiation and organismal functioning when lipogenesis is not inhibited. Therefore, adipogenesis initiation requires a basal level of lipogenesis, but thereafter lipogenesis can be uncoupled from adipocyte identity and functioning while inhibiting DGATs to only abolish TG synthesis and LD formation is not requisites for adipogenesis [31,32,48]. 

Samples from starved mice demonstrate that LD-deprived adipocytes with significantly reduced lipid synthetic and adipogenic gene expression still maintain lipid synthesis function after refeeding. This finding suggests that the presence of LDs and lipid synthetic and adipogenic gene activity can be uncoupled from adipocyte identity, likely under a natural state of nutrient scarcity-induced “dormancy.” The rapid response of lipid synthetic genes to refeeding supports lipid synthesis as being an inherent capacity of AT and confirms the centrality of AT as the main nutrient response and storage organ in the body. However, the maintenance of adipokine gene expression even under a nutrient-deprived state suggests that endocrine functioning may be more fundamental to adipocyte functioning than lipid storage. Therefore, a new conception of adipocyte identity might focus on the adipocyte as being an interpreter and communicator of many intra- and extra-cellular energy status signals, with lipid storage as a subservient feature of this activity. Furthermore, these signals together may determine whether conditions are optimal for new adipocyte formation, identity maintenance, or reverting into a dedifferentiated state, which may vary between depots. 

Beyond adipocytes, the function of lipid synthesis is also shared with non-adipocytes, such as hepatocytes and skeletal muscle cells [55,56]. Ectopic fat storage is commonly found in organs (e.g., heart, liver, and skeletal muscle) under metabolic dysfunctions in both humans and mice [51,52]. Clearly, lipid storage or LD presence is not a feature unique to adipocytes and, therefore, is not a defining feature of adipocytes. This is also true for dedifferentiated adipocytes, which may retain large amounts of LDs but lose adipocyte identity in terms of adipogenic, lipogenic, and adipokine gene expression. In this regard, the simplified narrative of AT as a lipid storage organ has obfuscated a nuanced understanding of adipocyte identity and function. The preferential storage of lipids in AT may be to protect other tissues from lipotoxicity. However, during conditions of overnutrition, the resulting dysfunctional AT expansion may become pathologic to AT and, thus, the organism as a whole.

The balance of AT expansion toward hypertrophic versus hyperplastic is more strongly associated than BMI with obesity complications, such as adipocyte dysfunction, ectopic lipid deposition, and early onset of insulin resistance. The protective mode of hyperplasia involves increasing adipogenesis and the number of adipocytes, while the detrimental mode of hypertrophy involves increasing lipid synthesis and the size of pre-existing adipocytes. Therefore, uncoupling lipid synthesis from adipocyte development shows promising potential as an application in obesity management. This uncoupling likely does not cause ectopic lipid deposition and metabolic detriments, given that basal de novo lipogenesis is maintained to sustain adipogenesis and other adipocyte functions, as shown in conditional adipocyte knockout of FASN and DGAT1/2 mouse models [31,35,47]. By uncoupling lipid synthesis from adipogenesis, we may shift the balance toward hyperplasia and away from hypertrophy to generate more metabolic healthy adipocytes and maintain the function of pre-existing adipocytes. Encouragingly, we recently achieved this uncoupling by using cationic nanomaterials to inhibit adipocyte hypertrophy while promoting adipogenesis in DIO mice, resulting in improved metabolic health [42,57]. Thus, uncoupling lipid synthesis from adipocyte development may foreseeably aid in combating obesity and its comorbidities.

## Data Availability

All study data are included in the article and Supplementary.

## Supplementary Materials

The following supporting information can be downloaded at: https://www.mdpi.com/article/10.3390/biomedicines11041132/s1, Figure S1: Cell morphology of liver and Brown adipose tissue (BAT) did not change in response to prolonged fasting; Figure S2: Biotin deprivation in 3T3-L1 preadipocyte differentiation; Figure S3: Inhibition of FASN shows stage-dependent effect on 3T3-L1 adipogenesis; Figure S4: Inhibiting TG synthesis does not block 3T3-L1 adipocyte differentiation despite prevent- ing lipid droplet accumulation; Table S1: List of primers used in QPCR.
