# Supplementary material for: Uncoupling Lipid Synthesis from Adipocyte Development
Source: Biomedicines. 2023 Apr 9;11(4):1132. doi: 10.3390/biomedicines11041132 (PMC10135928; doi:10.3390/biomedicines11041132)
Supplement: Supplementary file 1 [file biomedicines-11-01132-s001.zip › biomedicines-2280207-supplementary.pdf]

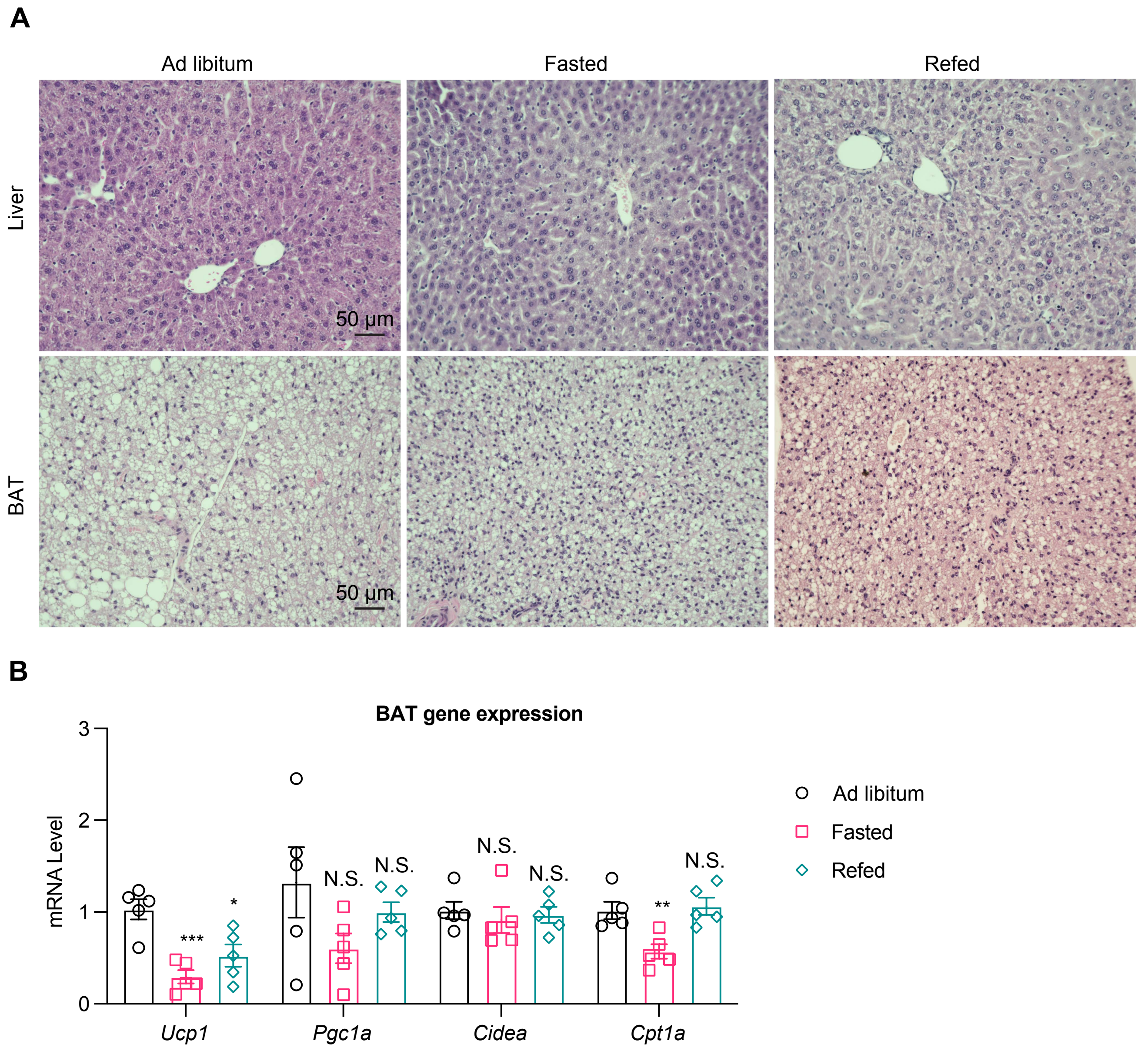

Supplementary Figure S1. Cell morphology of liver and Brown adipose tissue (BAT) did not change in response to prolonged fasting. A: H&E staining of BAT and liver from ad libitum fed, fasted mice or fast-ed-refed mice. B: qPCR analysis of gene expression of brown adipocyte markers in iWAT from ad libitum fed, fasted mice or fasted-refed mice. n=5/group. Data were represented as mean ± SEM. Statistical significance is calculated via a two-tailed Student's t-test (Fasted or refed group versus Ad libitum group). N.S.: not significant, *p < 0.05, ** p < 0.01, *** p < 0.001.

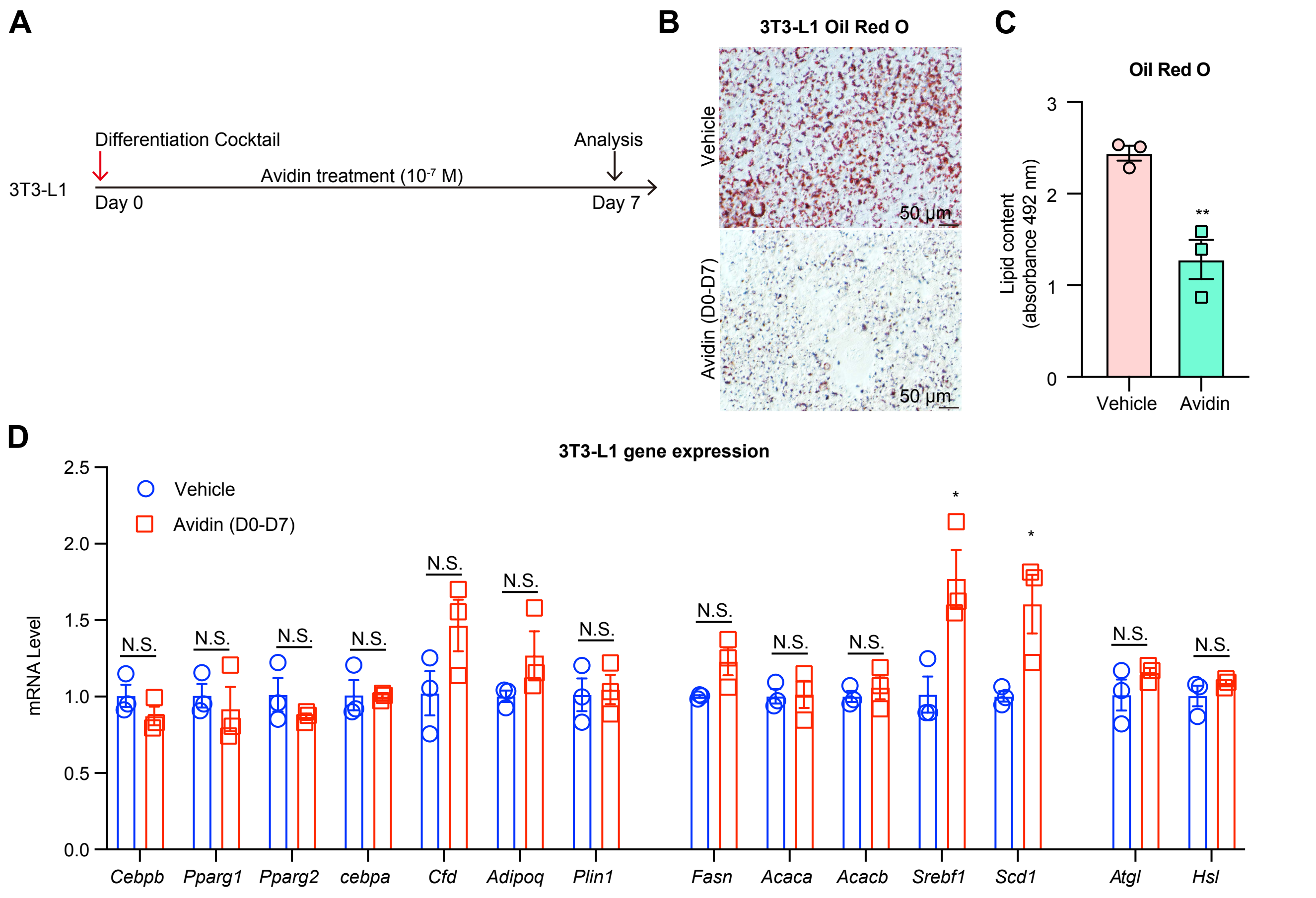

Supplementary Figure S2. Biotin deprivation in 3T3-L1 preadipocyte differentiation. A: Experimental design. 3T3-L1 preadipocytes were differentiated in the presence of 10^{-7} M Avidin from Day 0 to Day 7 of differentiation. B: Oil Red O staining of lipid droplets in 3T3-L1 cells on Day 7 of differentiation. C: Quantification of Oil Red O staining. D: qPCR analysis of gene expression of adipogenic markers and lipogenic genes on Day 7 of differentiation with or without Avidin treatment. (n=3, 3). N.S.: not significant, * $p < 0.05$, ** $p < 0.01$, for control vs. Avidin treatment group by 2-tailed Student's t-test. Data were represented as mean ± SEM.

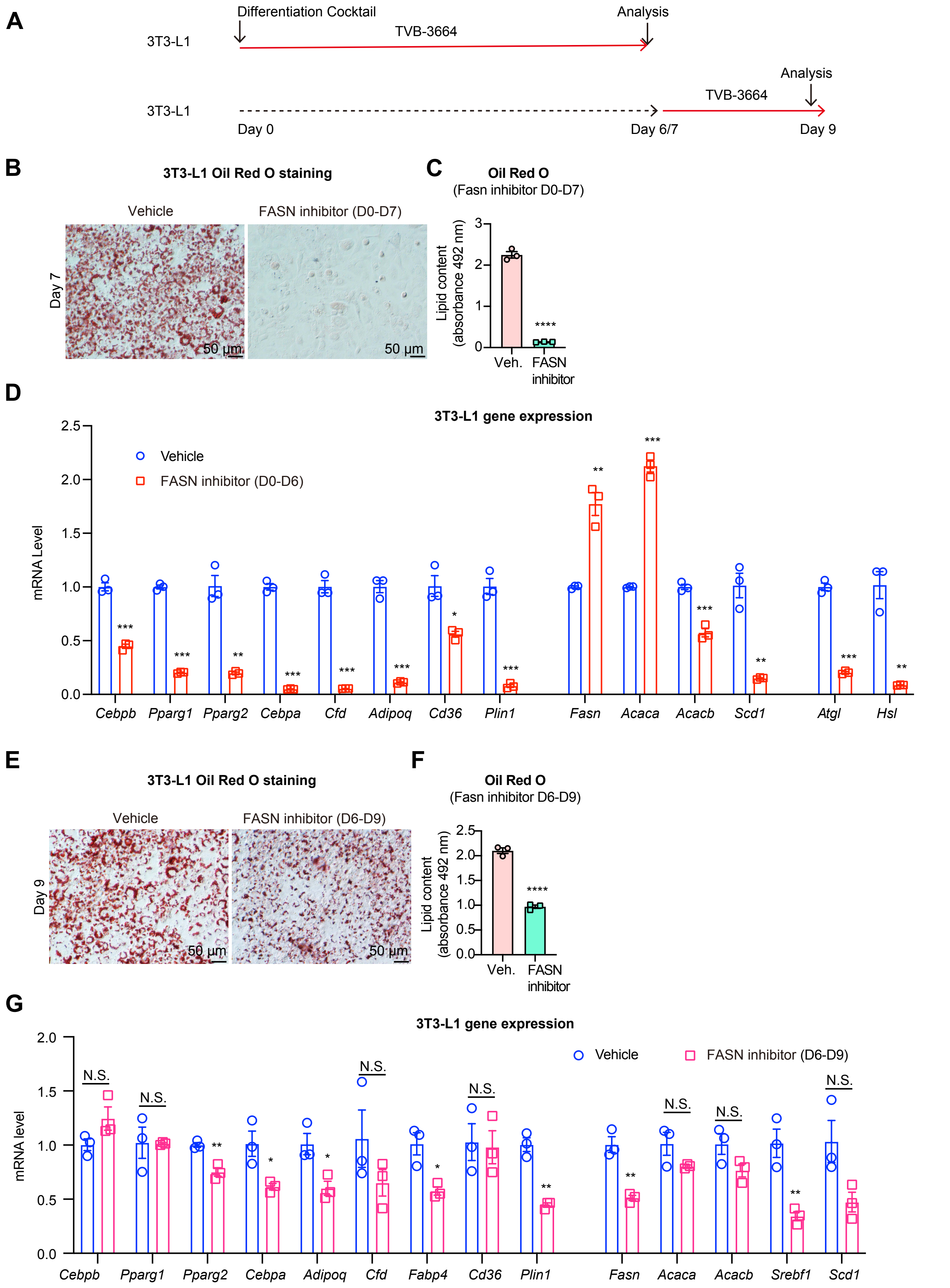

Supplementary Figure S3. Inhibition of FASN shows stage-dependent effect on 3T3-L1 adipogenesis. A: Experimental design. 3T3-L1 preadipocytes were treated with FASN inhibitor, TVB-3664 (200 nM) through differentiation Day 0 to Day 6/7, or mature 3T3-L1 cells were treated with FASN inhibitor from differentiation Day 6 to Day 9. B,C: Oil Red O staining of lipid droplets in 3T3-L1 cells (fixed on Day 7) with or without TVB-3664 treatment. D: Gene expression of adipogenic and lipogenic marker genes in 3T3-L1 cells treated with TVB-3664 at Day 0 to Day 6 during differentiation. E,F: Oil Red O staining of lipid droplets in 3T3-L1 cells (fixed on Day 9) with or without TVB-3664 treatment. G: Gene expression of adipogenic and lipogenic marker genes in 3T3-L1 cells treated with TVB-3664 at Day 6 to Day 9 during differentiation. n=3, 3. N.S.: not significant, * $p < 0.05$, ** $p < 0.01$, *** $p < 0.001$ for control vs. TVB-3664 treatment group by 2-tailed Student's t-test. Data were represented as mean ± SEM.

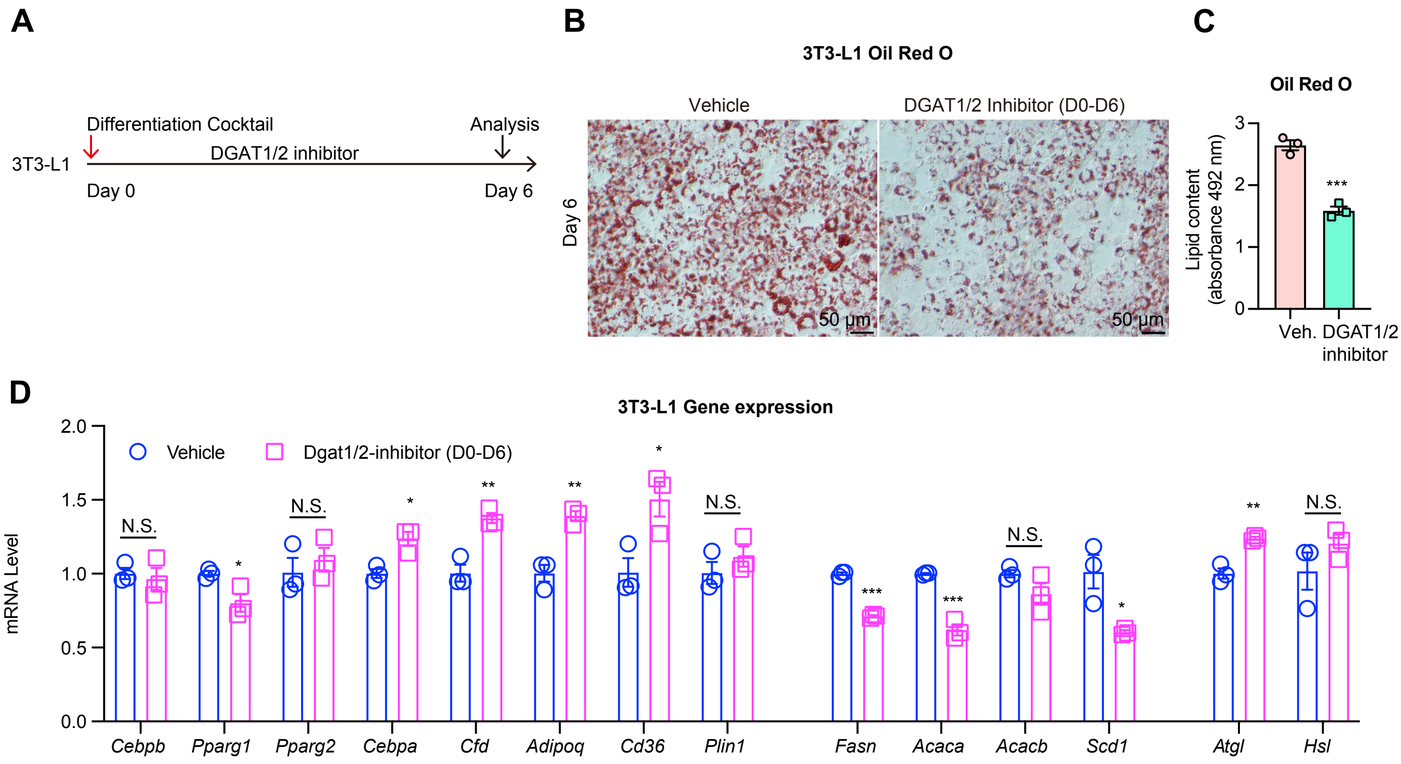

Supplementary Figure S4. Inhibiting TG synthesis does not block 3T3-L1 adipocyte differentiation despite preventing lipid droplet accumulation. A: Experimental design. 3T3-L1 preadipocytes were differentiated in the presence of DGAT1 inhibitor (PF-04620110, 3 μM) and DGAT2 inhibitor (PF-06427878, 3 μM) from Day 0 to Day 6. B,C: Oil Red O staining of lipid droplets in 3T3-L1 cells on Day 6 of differentiation. D: qPCR analysis of gene expression of adipogenic, lipogenic and lipolytic marker genes on Day 6 of differentiation. n=3, 3. N.S.: not significant, * $p < 0.05$, ** $p < 0.01$, *** $p < 0.001$ for control vs. DGAT1/2 inhibitor treatment group by 2-tailed Student's t-test. Data were represented as mean ± SEM.

Supplementary Table S1. List of primers used in QPCR.

Primer name	Primer sequence
Adipsin-F	CATGCTCGGCCCTACATGG
Adipsin-R	CACAGAGTCGTCATCCGTCAC
Perilipin 1-F	GGCCTGGACGACAAAACC
Perilipin 1-R	CAGGATGGGCTCCATGAC
Scd1-F	CATCATTCTCATGGTCCTGCT
Scd1-R	CCCAGTCGTACACGTCATTTT
Fasn-F	CTGACTCGGCTACTGACACG
Fasn-R	TGAGCTGGGTTAGGGTAGGA
Cd36-F	TCCAGCCAATGCCTTTGC
Cd36-R	TGGAGAATTACTTTTTCAGTGCAGAA
Srebf1-F	GAAGCTGTCGGGGTAGCGTCT
Srebf1-R	CTCTCAGGAGAGTTGGCACCTG
Acaca-F	ACACCATGTTGGGAGTTGTG
Acaca-R	GCTGTTCCTCAGGCTCACAT
Acacb-F	CATGGTAGTGGCTTTGAAGGA
Acacb-R	CGTGTCGATATCGTTGTTCTG
Pparg2-F	TCTGGGAGATTCTCCTGTTGA
Pparg2-R	GGTGGGCCAGAATGGCATCT
Pparg1-F	AGAAGCGGTGAACCACTGAT
Pparg1-R	GAATGCGAGTGGTCTTCCAT
Cebpa-F	GGACAAGAACAGCAACGAGTA
Cebpa-R	GCAGTTGCCATGGCCTTGA
Cebpb-F	CAAGCTGAGCGACGAGTACA
Cebpb-R	CAGCTGCTCCACCTTCTTCT
Fabp4-F	AAGTGGGAGTGGGCTTTGC
Fabp4-R	CCGGATGGTGACCAAATCC
Adiponectin-F	GCACTGGCAAGTTCTACTGCAA
Adiponectin-R	GTAGGTGAAGAGAACGGCCTTGT
Dgat2-F	AGTGGCAATGCTATCATCATCGT
Dgat2-R	TCTTCTGGACCCATCGGCCCCAGGA
Agpat2-F	GCAACGACAATGGGGACCTG
Agpat2-R	ACAGCATCCAGCACTTGTACC
Gpat3-F	GGAGGATGAAGTGACCCAGA

Gpat3-R	CCAGTTTTTGAGGCTGCTGT
Ucp1-F	ACTGCCACACCTCCAGTCATT
Ucp1-R	CTTTGCCTCACTCAGGATTGG
Atgl-F	AACACCAGCATCCAGTTCAA
Atgl-R	GGTTCAGTAGGCCATTCCTC
Cidea-F	TGCTCTTCTGTATCGCCCAGT
Cidea-R	GCCGTGTTAAGGAATCTGCTG
Cpt1a-F	TGCACTACGGAGTCCTGCAA
Cpt1a-R	GGACAACCTCCATGGCTCAG
Hsl-F	ACCGAGACAGGCCTCAGTGTG
Hsl-F	GAATCGGCCACCGGTAAAGAG
Cyclophilin A-F	TATCTGCACTGCCAAGACTGAGTG
Cyclophilin A-R	CTTCTTGCTGGTCTTGCCATTCC
Rpl23-F	CTGTGAAGGGAATCAAGGGA
Rpl23-R	TGTCGAATTACCACTGCTGG
